# A novel elicitor protein phosphopentomutase from *Bacillus velezensis* LJ02 enhances tomato resistance to *Botrytis cinerea*

**DOI:** 10.3389/fpls.2022.1064589

**Published:** 2022-11-29

**Authors:** Zhuoran Li, Jianan Hu, Qi Sun, Xi Zhang, Ruokui Chang, Yuanhong Wang

**Affiliations:** ^1^ College of Horticulture and Landscape Architecture, Tianjin Agricultural University, Tianjin, China; ^2^ College of Engineering and Technology Architecture, Tianjin Agricultural University, Tianjin, China

**Keywords:** Bacillus velezensis LJ02, phosphopentomutase, tomato disease resistance, Botrytis cinerea, immune response, signaling pathway

## Abstract

The loss of tomatoes caused by *Botrytis cinerea* (*B. cinerea*) is one of the crucial issues restricting the tomato yield. This study screened the elicitor protein phosphopentomutase from *Bacillus velezensis* LJ02 (BvEP) which improves the tomato resistance to *B. cinerea*. Phosphatemutase was reported to play a crucial role in the nucleoside synthesis of various microorganisms. However, there is no report on improving plant resistance by phosphopentomutase, and the related signaling pathway in the immune response has not been elucidated. High purity recombinant BvEP protein have no direct inhibitory effect on *B. cinerea in vitro*,and but induce the hypersensitivity response (HR) in *Nicotiana tabacum*. Tomato leaves overexpressing BvEP were found to be significantly more resistant to *B. cinerea* by *Agrobacterium*-mediated genetic transformation. Several defense genes, including *WRKY28* and *PTI5* of PAMP-triggered immunity (PTI), *UDP* and *UDP1* of effector-triggered immunity (ETI), *Hin1* and *HSR203J* of HR, *PR1a* of systemic acquired resistance (SAR) and the SAR related gene *NPR1* were all up-regulated in transgenic tomato leaves overexpressing BvEP. In addition, it was found that transient overexpression of BvEP reduced the rotting rate and lesion diameter of tomato fruits caused by *B. cinerea*, and increased the expression of PTI, ETI, SAR-related genes, ROS content, SOD and POD activities in tomato fruits, while there was no significant effect on the weight loss and TSS, TA and Vc contents of tomato fruits. This study provides new insights into innovative breeding of tomato disease resistance and has great significance for loss reduction and income enhancement in the tomato industry.

## Introduction

Grey mold is one of the fungal diseases causing the most serious economic losses in the tomato industry (Dean et al., 2012; Abbey et al., 2019). *Botrytis cinerea* (*B. cinerea*), as a plant necrotrophic pathogen causing grey mold, can regulate antagonism between immune pathways to promote the development of the disease (El Oirdi et al., 2011). The elicitor protein produced by beneficial or pathogenic microorganisms can enhance tomato resistance to *B. cinerea* by inducing plant immune responses. The protein elicitor PebC1 isolated and purified from the mycelium of *B. cinerea* can induce the disease resistance of tomato to *B. cinerea* and increase the activity of enzymes related to plant metabolisms such as phenylalanine ammonia-lyase (PAL), peroxides (POD), and polyphenol oxidase (PPO) (Zhang et al., 2010). The elicitor BcGs1, a necrosis inducing protein purified from the culture filtrate of *B. cinerea*, can elicit resistance to *B. cinerea* in tomato leaves and the transcript levels of the defense-related genes *PR-1a*, *TPK1b* and *Prosystemin* (Zhang et al., 2015). The oli-D1 and oli-D2 cytokine receptor protein from *Pythium Oligandrum* can induce a hypersensitive response (HR) in *Nicotiana benthamiana* and induce resistance to *B. cinerea* in tomato leaves (Ouyang et al., 2015). The Oligandrin protein of *Pythiumo ligandrum* can significantly increase resistance to *B. cinerea*, and induced the expression levels of genes *PR-2a*, *PR-3a*, *LeERF2*, *PR6* and the activities of PAL, PPO and POD enzymes in tomato fruits (Wang et al., 2011). Chitin isolated from yeast cell wall can induce the resistance of tomato fruit to *B. cinerea* and promote the accumulation of ROS, moreover, the activities of superoxide dismutase (SOD), POD enzymes and the expression of these corresponding genes, the key enzyme genes of SA biosynthesis and signal transduction pathway were increased in the chitin-treated fruit (Zhu and Zhang, 2016).

Elicitors can induce plants to activate two complex and efficient immune systems, pathogen-associated molecular patterns/microbial-associated molecular patterns (PAMPs/MAMPs) triggered immunity (PTI/MTI), and can induce up-regulated expression of the *WRKY28* and *PTI5* genes, resisting the infection of various pathogens (Tran et al., 2017; Abdul Malik et al., 2020; Ngou et al., 2021). Plants also produce resistance proteins (such as R proteins) to recognize effectors produced by specific pathogenic microorganisms that inhibit PTI/MTI, resulting in effector-triggered immunity (ETI) defense responses (Ngou et al., 2021). The *UDP* and *UDP1* genes were induced only during ETI (Pombo et al., 2014; Chialva et al., 2018). ETI usually accompanied by hypersensitive response (HR), resulting in production of the resistance to exogenous pathogens (Zebell and Dong, 2015). In addition, PTI/MTI and ETI generate immune signals at the site of infection, which in turn activates systemic acquired resistance (SAR) in uninfected tissues of plants to resist further attack by pathogens (Tian and Zhang, 2019). Up-regulated expression of *PR1* genes such as *PR1a* is one of the markers of SAR (Wang et al., 2015; Liu et al., 2019). SA signaling is transduced by the *NPR1* protein, which acts as a transcriptional co-activator of numerous PR genes and is required for PR gene expression, SA signaling, and SAR production (Wu et al., 2012; Wang et al., 2018). The production of PTI/MTI, ETI and SAR responses is accompanied by a series of changes such as the generation of reactive oxygen species (ROS) burst and activation of the mitogen-activated protein kinase (MAPK) signaling pathway in plant cells, which in turn induces transcriptional reprogramming of defense gene expression to resist pathogens (Ngou et al., 2022).

Phosphopentomutase (PPM) is found in bacterial and mammalian cells and is responsible for the interconversion of ribose-5-phosphate (R5P) and ribose-1-phosphate (R1P). R5P is the direct precursor of 5-phosphoribosyl-1-pyrophosphate (PRPP), and PRPP is used for nucleotide synthesis (Rashid et al., 2004; Panosian et al., 2011). PPM transfers the phosphate group from the C5 position of R5P to the C1 position, generating R1P, thus linking glucose metabolism and RNA biosynthesis (Rashid et al., 2004; Moustafa et al., 2016). R1P acts as a substrate for purine or pyrimidine nucleoside phosphorylase (PNP or PyNP), which replaces phosphate and form nucleoside (Tozzi et al., 2006). Furthermore, PPM also catalyzes the transfer of intramolecular phosphate from C1 to C5 in ribose, participates in pentose phosphate pathway and purine metabolism to obtain a source of carbon and energy, and plays an important role in nucleoside synthesis of various microorganisms (Rashid et al., 2004; Tozzi et al., 2006). However, there is no report on PPM enhancing plant immunity and disease resistance.

*Bacillus velezensis* (*B.velezensis*) LJ02, as a beneficial microorganism, can enhance plant resistance to *B. cinerea* and other pathogens, and can also secrete elicitor proteins to induce plant immune responses (Li et al., 2015; Hu et al., 2022). In this study, the new elicitor protein BvEP, which can improve the control effect of tomatoes on *B. cinerea*, was selected from *B. velezensis* LJ02. The potential mechanism that BvEP enhanced the control effect of tomatoes to *B. cinerea* was explored. BvEP protein was obtained by prokaryotic expression and purification. The direct antibacterial effect of BvEP protein on *B. cinerea in vitro* was excluded, and it was proved that BvEP could activate the plant immune response by the HR on *Nicotiana tabacum*. Transgenic tomato plants overexpressing BvEP could significantly improve the resistance to *B. cinerea*, and the resistance-related signaling pathway was up-regulated through gene expression analyses in transgenic tomatoes. Furthermore, the effect of BvEP on disease resistance of tomato fruit was verified by transient expression, BvEP overexpression could improve the resistance of tomato fruits to *B. cinerea*, and increase the content of ROS, the activities of SOD and POD enzyme, and the expression of resistance related genes, however, the transient overexpression of BvEP did not significantly affect the weight loss rate and TSS, TA and Vc content of tomato fruits.

## Materials and methods

### Plant, bacteria and pathogens culture conditions

Tobacco plants (*Nicotiana tabacum*) and Tomato plants (*Lycopersicum esculentum Mill*) were grown at 25°C, with 16 h light/8 h dark cycle in a phytotron. Cherry tomato (*Lycopersicon esculentum* var. *cerasiforme*) fruits were harvested for subsequent experiments. *B.velezensis* LJ02 was grown on Luria-Bertani (LB) medium at 37°C (Li et al., 2015). *B. cinerea* was cultivated on potato dextrose agar(PDA)medium at 25°C.

### Verification of *B. velezensis* LJ02 immune clone

The clones corresponding to the Fosmid fermentation library were shaken at 37°C, the bacteria supernatant was infiltrated into the tomato leaves with a 1 mL needleless syringe. The systemic leaves of tomato were collected and inoculated with *B. cinerea* mycelium discs at 2 days after infiltration. The diameter of lesions on tomato leaves was measured by the criss-cross method (Hu et al., 2022) and photographed at 3 days after inoculation (DAI).

### Construction of BvEP overexpression vector and transient expression

The LJ02 genome DNA was extracted using a bacterial genomic DNA extraction kit (Solarbio). The LJ02 genome DNA was used as the template to amplify the BvEP DNA fragment, and the BvEP DNA fragment was cloned into the overexpression vector pK7LIC4.0 (expressed by 35S promoter) by homologous recombination (Vazyme). The constructed vectors were transferred into *Agrobacterium* GV3101. GV3101 containing pK7LIC4.0-BvEP was infiltrated into tomato leaves, the infiltration method was according to method described previously (Li et al., 2019).

### BvEP protein prokaryotic expression and purification

The BvEP DNA fragment was cloned into the prokaryotic vector pET30a by homologous recombination. The constructed vectors were transferred into *E. coli* BL21. The competent cell containing pET30a-BvEP were cultured to OD_600_ of 0.6, isopro-pyl-β-d-mercaptogalactoside (IPTG) was added to a final concentration of 0.1 mM, and expression was induced at 16°C for 12 h. After centrifugation, the cells were resuspended by lysis buffer (1 mol/L Tris-HCl (pH 8.0); 4 mol/L NaCl; glycerol (w/v) 10%) and disrupted by ultrasonication. The supernatant was added to Ni Sepharose (GE) for purification. After purification, the purified protein was filtered by 0.22 um filter membrane. 100 μL of purified protein was detected and analyzed by SDS-PAGE and BSA was used as standard substance, then the separation gel was stained with Coomassie brilliant blue fast staining solution (Solarbio). The concentration was determined by Bradford method and recorded. The protein expression, purification and detection method was described previously (Hu et al., 2022).

### Effects of BvEP on *B. cinerea in vitro*

Antifungal activity of BvEP was evaluated by the oxford cup method, which was used to observe bacteriostatic circle occurred near the oxford cup. The method was described previously (Li et al., 2013). The sterilized oxford cup and the mycelial discs of *B. cinerea* were placed symmetrically on the PDA solid medium plate. 100 μL of BvEP purified protein was added to the oxford cup, and the Oxford cup added with protein buffer was used as a control. The PDA medium plate was cultured at 25°C for 3 d.

The spore germination rate of *B. cinerea* was measured to evaluate the BvEP antibacterial activity. 10 μL of spore suspension (1 × 10^8^ conidia/ml) and 10 μL of purified BvEP protein were dropped on a glass slide, the glass slide with spore suspension and protein buffer were used as a control. The glass slide was cultured at 28°C for 12 h, and the number of spore germinations was calculated under a microscope. Spores germination assays and calculation of its inhibition rate according to the method described previously (Plascencia-Jatomea et al., 2003; Zhao et al., 2014).

### Verification of HR on *Nicotiana tabacum*

The method of HR verification was described previously (Wang et al., 2016). A needle-free syringe was used to infiltrate BvEP purified protein into *Nicotiana tabacum* leaf. HR symptoms were observed at the infiltration site at 24 h after infiltration.

### Genetic transformation of tomato

The *Agrobacterium* GV3101 transformed into pK7LIC4.0-BvEP was genetically transformed into tomato (*Solanum lycopersicum* A57), and the transgenic A57 genetically transformed with the empty vector pK7LIC4.0 was used as a control. The transformation process was described previously (Sun et al., 2006). Reverse transcription PCR (RT-PCR) was used to detect the mRNA expression of BvEP in transgenic tomato plants. The primer sequences used for identification of transgenic tomatoes were shown in **Supplementary Table 1**.

### Quantitative real-time PCR

The specific primers sequence for *PTI5*, *WRKY28*, *PR1a* and *NPR1* in tomato leaves and fruits described previously (Liu et al., 2012; Sun et al., 2013; Chialva et al., 2018; Zhang et al., 2020a). The sequences of specific primers for *UDP* and *UDP1* in tomato fruits were described previously (Pombo et al., 2014). Actin sequence was used as the internal reference gene (Zhang et al., 2020a). The specific primers sequence for *UDP* and *UDP1* in tomato leaves were designed by using reference GenBank SnapGene software (www.ncbi.nlm.nih.gov/genbank/) in the database of tomato (**Supplementary Table 2**). RNA was extracted from tomato leaf samples and reverse transcription was performed. Real-time PCR (RT-PCR) was used to verify the expression pattern of candidate genes. The data calculation method used the 2^-ΔΔCt^ method. Methods for quantitative real-time PCR and analysis were previously described (Livak and Schmittgen, 2001).

### Transient transformation of tomato fruits

The *Agrobacterium* GV3101 transformed into pK7LIC4.0-BvEP was transient transformed into cherry tomato fruits, and the fruits which transiently transformed with the empty vector pK7LIC4.0 were used as control. Tomato fruits were infiltrated with 100 uL of pK7LIC4.0-BvEP *Agrobacterium* cultures. The transformation process was described previously (Orzaez et al., 2006). Reverse transcription PCR (RT-PCR) was used to detect the mRNA expression of BvEP in tomato fruit. The primer sequences used for identification of transient tomato fruit were shown in **Supplementary Table 1**.

### Inoculation of *B. cinerea* on tomato leaves and fruits

The tomato leaves were inoculated with *B. cinerea* mycelium disc, the inoculation method was described previously (Wang et al., 2016). The leaves were placed in a plate containing wet filter paper to maintain high-humidity, then the plate was placed in a phytotron at 25°C for 3 d. The lesion diameter was measured (Hu et al., 2022) and photographed at 3 DAI.

The method of inoculation on tomato fruits was described previously (Sun et al., 2018). The tomato fruit was inoculated with 20 μL of *B. cinerea* spore suspension (1 × 10^6^ conidia/ml) on the equatorial surface. The rotting rate and lesion diameter were calculated according to the method of Zhao et al. (2008). Three biological replicates were determined for each sample, and 15 tomato fruits were used as one biological replicate.

### ROS and its related enzyme activities assay

ROS content and SOD, POD activities in tomato fruits of transient transformation with pK7LIC4.0 and pK7LIC4.0-BvEP was determined. The tomato fruits were ground into powder with liquid nitrogen, 0.1g powder was weighed for the above index determination. Three biological replicates were determined for each sample, 5 tomato fruits were used as one biological replicate.

The ROS detection method was described previously (Hu et al., 2022). The ROS content in the fruits was determined by adding 0.1 g of tomato fruit powder to phosphate buffered saline (pH 7.4) followed by a commercial plant ROS enzyme-linked immunosorbent assay (ELISA) kit (Chundubio).

Methods for SOD and POD activities assay were also described previously (He et al., 2019). 0.1g of tomato fruit powder was added to 1 mL of the extraction solution, centrifuged at 8000g for 10min at 4°C, and the supernatant was taken on ice for determination. SOD activity was measured with the SOD enzyme activity assay kit (Solarbio) by UV spectrophotometer with absorbance at the maximum absorption wavelength of 470 nm. POD activity was measured with the POD enzyme activity assay kit (Solarbio) by UV spectrophotometer with absorbance at the maximum absorption wavelength of 560 nm.

### Fruit quality determination

Weight loss rate and total soluble solids (TSS), titratable acidity (TA), vitamin C (Vc) content of tomato fruits of transient transformation with pK7LIC4.0 and pK7LIC4.0-BvEP was determined. Three biological replicates were determined for each sample, 10 tomato fruits were used as one biological replicate. The determination of tomato fruit weight loss was described previously (Wu et al., 2022). Methods for TSS, TA, Vc content were also described previously (Sdiri et al., 2012).

## Results

### Tomato treated with 1-1F5 can improve the control effect to *B. cinerea*

**Figure 1:**
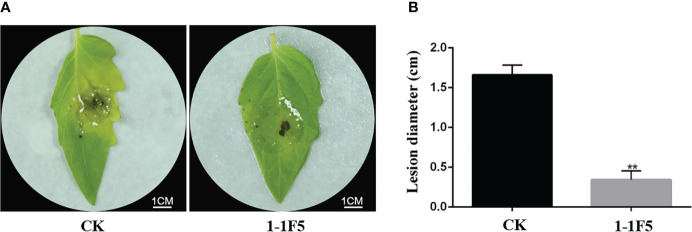
Effects of 1-1F5 on tomato leaves inoculated with *B.cinerea*. **(A)** Phenotype of the systemic leaves of tomato at 3 DAI with *B. cinerea*. CK and 1-1F5 are respectively the inoculation of *B. cinerea* after the infiltration of ddH_2_O and 1-1F5 supernatant into tomato leaves; **(B)** Lesion diameter of tomato at 3 DAI with *B. cinerea*, asterisks indicate statistically significant differences (** *p* < 0.01).

To screen the immune clone that can improve the control effect of tomato against *B. cinerea* from LJ02, the Fosmid library of LJ02 was constructed by using *E. coli* EPI300-TIR as the host strain, and the corresponding clones were cultured at 37°C centrifuged with ddH_2_O. The resuspended clone was infiltrated into tomato leaves, and the systemic leaves above the tomato were harvested after 2 days to inoculate *B. cinerea*. The tomato systemic leaves collected 2 days after ddH_2_O infiltration were inoculated with *B. cinerea* as the control group (CK). The results showed that 3DAI with *B. cinerea*, severe symptoms were observed on the leaves of CK, while the symptoms on the leaves of the immune clone 1-1F5 were mild (**Figure 1A**). Moreover, the lesion diameter on the leaves of 1-1F5 was significantly lower than CK (**Figure 1B**), indicating that the immune clone 1-1F5 had a significant effect on improving the control effect of tomato against *B. cinerea*.

### BvEP can improve the control effect of tomato leaves to *B. cinerea*

**Figure 2:**
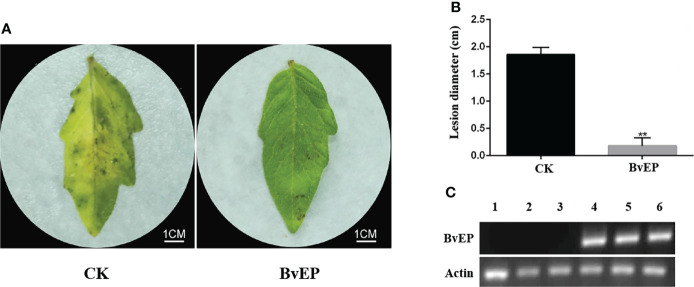
Effects of BvEP transient transformation on tomato leaves after inoculation with *B. cinerea*. **(A)** Phenotype of tomato systemic leaves inoculation at 3 DAI with *B.cinerea*; leaves were treated as follows before inoculation: CK represented pK7LIC4.0 infiltration, BvEP represented pK7LIC4.0-BvEP infiltration; **(B)** Lesion diameter of tomato at 3 DAI with *B. cinerea*, asterisks indicate statistically significant differences (** *p* < 0.01). **(C)** Expression of BvEP mRNA in tomato leaves at 3 DAT with pK7LIC4.0 and pK7LIC4.0-BvEP; 1, 2, 3 are pK7LIC4.0; 4, 5 and 6 are pK7LIC4.0-BvEP.

The immune clone 1-1F5 was sequenced, and the BvEP sequence of the complete insert was obtained. The BvEP was cloned and constructed into the overexpression vector pK7LIC4.0 containing the 35S promoter for transient transformation of tomato leaves, and *B. cinerea* was inoculated on the tomato systemic leaves at 3 days after transient transformation (DAT). Tomato systemic leaves inoculated with *B. cinerea* at 3 DAT with pK7LIC4.0 were used as CK. RT-PCR analysis of tomato leaves showed that the BvEP mRNA was stably accumulated at 3 DAT (**Figure 2C**). A mild symptom was observed on the transient transformation of tomato systemic leaves with BvEP compared to CK 3 DAI (**Figure 2A**), and the lesion diameter on the tomato systemic leaves was significantly smaller than CK (**Figure 2B**).

### BvEP has no direct inhibitory effect on *B. cinerea*, and but induce HR on *Nicotiana tabacum*

**Figure 3:**
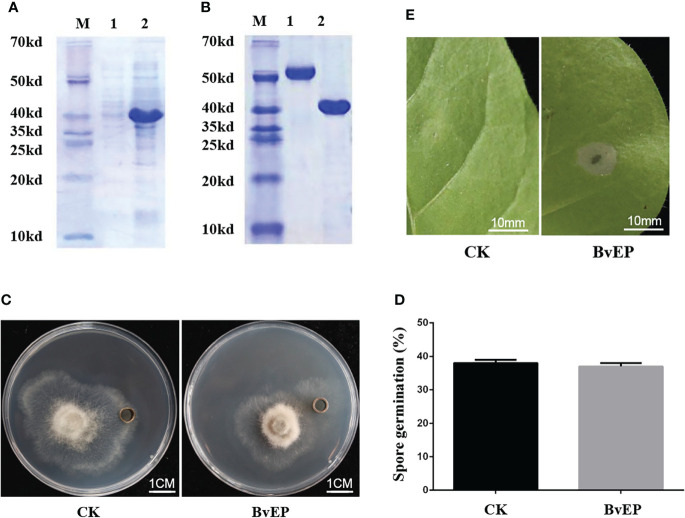
Inhibitory effect of BvEP protein on *B. cinerea* and HR induction in *Nicotiana tabacum*. **(A)** SDS-page gel image of the protein after BvEP expression, 1 and 2 are BvEP before and after IPTG induction respectively; **(B)** SDS-page gel image of BvEP protein after purification, 1 and 2 are 1.50 μg BSA and purified BvEP protein respectively; **(C)** Inhibition experiment of BvEP on *B. cinerea in vitro*; Figures CK and BvEP are Tris buffer and 50 μg·mL^-1^ BvEP protein in oxford cup, respectively; **(D)** Effects of BvEP purified protein on spore germination of *B. cinerea*. **(E)** BvEP protein HR assay; HR of BvEP on *Nicotiana tabacum*; CK and BvEP indicate infiltration of Tris buffer and 50 μg·mL^-1^ BvEP protein, respectively.

To verify whether the BvEP protein has a direct inhibitory effect on *B. cinerea*, the prokaryotic expression vector pET30a-BvEP was constructed and transformed into *E. coli* BL21. The expression protein of BvEP was obtained (**Figure 3A**) and purified by nickel column and dissolved in Tris buffer (**Figure 3B**). The lesion diameter of the systemic leaves in tomato infiltrated with BvEP purified protein was significantly smaller than in tomato leaves infiltrated with Tris buffer (**Supplementary Figure 1**), similar to the transient expression results.

The purified BvEP protein was used for the antibacterial experiment against *B. cinerea in vitro*, and the Tris buffer without BvEP was used as the CK to determine the effect of BvEP on mycelium growth and spore germination of *B. cinerea*. The results showed that there was no inhibition zone around the oxford cup containing the BvEP purified protein and CK (**Figure 3C**), and there was no significant difference in the spore germination rate of *B. cinerea* treated with the BvEP purified protein compared to CK (**Figure 3D**), indicating that BvEP has no obvious inhibitory effect on the growth of hyphals and spore germination of *B. cinerea in vitro*.

In addition to the direct inhibitory effect on pathogens, the induction of plant immune responses as an inducer may be an important mechanism for BvEP to improve resistance to disease. To determine whether BvEP can induce a plant immune response, 100 μL of 50 μg/mL purified BvEP protein was infiltrated into *Nicotiana tabacum* leaves, and the leaves infiltrated with Tris buffer were used as CK. The HR was observed after 1days after infiltration. The results showed that necrotic spot of about 9 mm were formed around leaves infiltrated with BvEP protein, while the CK had no obvious symptoms (**Figure 3E**), indicating that BvEP could induce the immune response of *Nicotiana tabacum*.

### BvEP-overexpressed transgenic tomato can significantly improve the resistance to *B. cinerea*

**Figure 4:**
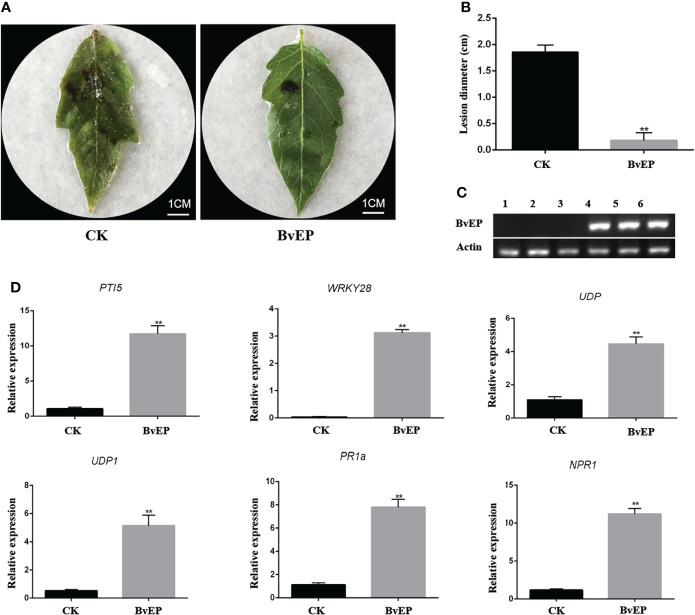
Resistance to *B. cinerea* and expression of disease-resistant genes in transgenic tomato leaves with BvEP overexpression. **(A)** Phenotype of genetically transformed tomato leaves at 3 DAI with *B. cinerea*, Figures CK and BvEP are pK7LIC4.0 and pK7LIC4.0-BvEP positive genetically transformed seedlings, respectively; **(B)** The lesion diameter on tomato leaves at 3DAI with *B. cinerea*, asterisks indicate statistically significant differences (** *p* < 0.01); **(C)** Expression of BvEP mRNA in transgenic tomato leaves; 1, 2, 3 are pK7LIC4.0 positive genetically transformed seedlings, 4, 5 and 6 are pK7LIC4.0-BvEP positive genetically transformed seedlings; **(D)** Expression levels of marker genes of resistance pathway in transgenic tomato leaves of pK7LIC4.0 and pK7LIC4.0-BvEP, asterisks indicate statistically significant differences (** *p* < 0.01).

PPM has no homologous gene expression in tomato, and there is no report on improving resistance to plant disease. To further verify the biological function and mechanism related to disease resistance of BvEP, its overexpression vector pK7LIC4.0-BvEP was genetically transformed into tomato cultivar A57. RT-PCR analysis of tomato leaves showed that BvEP mRNA could be stably accumulated in transgenic tomatoes (**Figure 4C**). BvEP transgenic tomato leaves were inoculated with *B. cinerea*, and the A57 tomato leaves transformed with pK7LIC4.0 were inoculated with *B. cinerea* as CK to verify the resistance of BvEP-overexpressed transgenic tomato leaves to *B. cinerea*. The results showed that the disease symptoms of the transgenic tomato leaves overexpressing BvEP were lighter than CK (**Figure 4A**), and the lesion diameter was significantly smaller than CK (**Figure 4B**).

BvEP can induce HR on *Nicotiana tabacum* (**Figure 3E**). To further explore the disease resistance signal pathway induced by BvEP in tomato, the expression of genes in the PTI, ETI and SAR resistance pathway such as *PTI5*, *WRKY28*, *UDP*, *UDP1*, *PR1a* and *NPR1* in transgenic tomato leaves overexpressed with BvEP was analyzed. The genetically transformed tomato leaves transformed with the pK7LIC4.0 were used as CK. The results showed that the expression levels of *PTI5*, *WRKY28*, *UDP*, *UDP1*, *PR1a* and *NPR1* in transgenic tomato leaves overexpressed with BvEP were significantly higher than in CK (**Figure 4D**).

### BvEP improves tomato fruit resistance to *B. cinerea*

**Figure 5:**
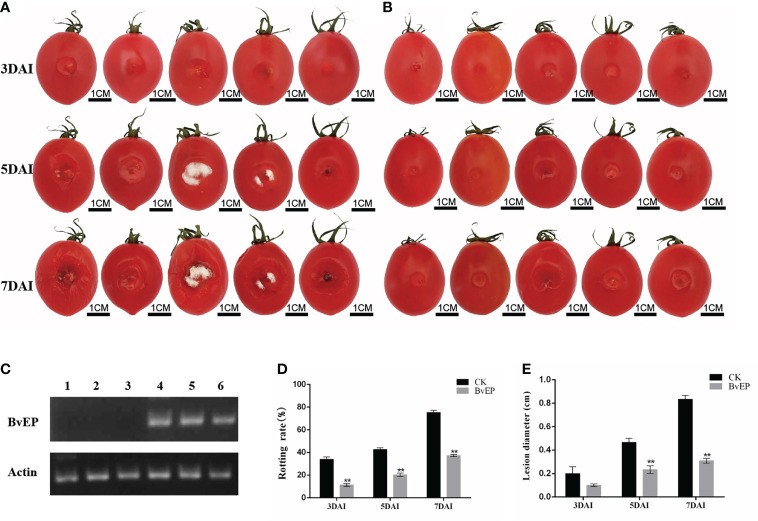
Effect of BvEP on resistance to *B. cinerea* of tomato fruits during storage. **(A)** The infection process of *B. cinerea* on tomato fruits after transient transformation with pK7LIC4.0; **(B)** Infection process of *B.cinerea* on tomato fruits after transient transformation with pK7LIC4.0-BvEP; **(C)** Expression of BvEP mRNA in tomato fruits at 3 DAT with pK7LIC4.0 and pK7LIC4.0-BvEP; 1, 2, 3 are pK7LIC4.0; 4, 5 and 6 are pK7LIC4.0-BvEP; **(D)** Rotting rate of tomato fruits at 3 DAI, 5 DAI, and 7 DAI with *B.cinerea*; **(E)** Lesion diameter on tomato fruits at 3·DAl, 5·DAl, and· 7·DAI with *B. cinerea*; asterisks indicate statistically significant differences (** p<0.01).

To explore the effect of BvEP on tomato fruit resistance to *B. cinerea*, cherry tomato fruits were transiently transformed with pK7LIC4.0-BvEP and inoculated with *B. cinerea* at 3 DAT. Tomato fruits inoculated with *B. cinerea* 3days after transient pK7LIC4.0 transformation were used as CK. A severe symptom caused by *B. cinerea* was observed on the tomato fruits of CK compared with transiently transformed by BvEP at 3 DAI, 5 DAI, and 7 DAI (**Figures 5A, B**). RT-PCR analysis of tomato fruits showed that the BvEP mRNA was stably accumulated at 3 days after BvEP transformation (**Figure 5C**). Tomato fruits transiently transformed by pK7LIC4.0-BvEP showed significantly lower rotting rate in 3 DAI, 5 DAI, and 7 DAI than CK (**Figure 5D**), and the lesion diameter was significantly lower in 5 DAI and 7 DAI than CK (**Figure 5E**).

### BvEP can significantly improve the resistance-related gene expression levels, the ROS accumulation and related enzyme activities in tomato fruit

**Figure 6:**
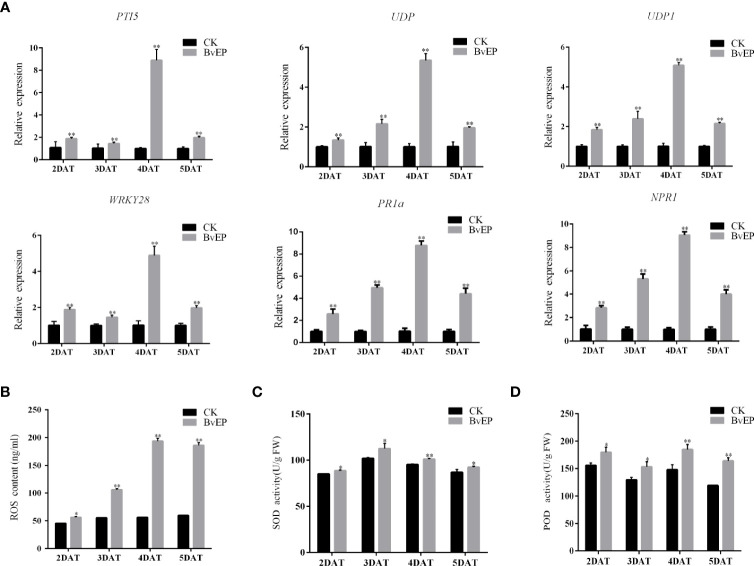
Effects of BvEP on resistance-related genes, ROS content and related enzyme activities in tomato fruits during storage. **(A)** Expression levels of marker genes of resistance pathways in tomato fruit after transient transformation with pK7LIC4.0 and pK7LIC4.0-BvEP; **(B)** ROS content in tomato fruit at 2 DAT, 3 DAT, 4 DAT, and 5 DAT with pK7LIC4.0 and pK7LIC4.0-BvEP; **(C, D)** are separately SOD and POD enzyme activities in tomato fruit at 2 DAT, 3 DAT, 4 DAT, and 5 DAT with pK7LIC4.0 and pK7LIC4.0-BvEP; asterisks indicate statistically significant differences (* *p* < 0.05, ** *p* < 0.01).

BvEP was able to significantly increase the expression of PTI, ETI, and SAR resistance-related genes in tomato leaves (**Figure 4D**). To further verify the effect of BvEP on resistance-related pathways in tomato fruits, the expression levels of *PTI5*, *WRKY28*, *UDP*, *UDP1*, *PR1a* and *NPR1* were also analyzed in tomato fruits transiently transformed with BvEP overexpression. The tomato fruits transformed with the pK7LIC4.0 were used as CK. The results showed that the expression of *PTI5*, *WRKY28*, *UDP*, *UDP1*, *PR1a* and *NPR1* in tomato fruits at 2 DAT, 3 DAT, 4 DAT, and 5 DAT of were significantly higher than CK (**Figure 6A**).

To further explore the effect of BvEP on disease resistance pathways in tomato fruits, the content of ROS and the activities of SOD and POD were measured. The results showed that the ROS content and activities of SOD and POD in tomato fruits at 2 DAT, 3 DAT, 4 DAT, and 5 DAT were significantly higher than CK (**Figures 6B-D**).

### BvEP has no significant effect on tomato fruit quality indexes

**Figure 7:**
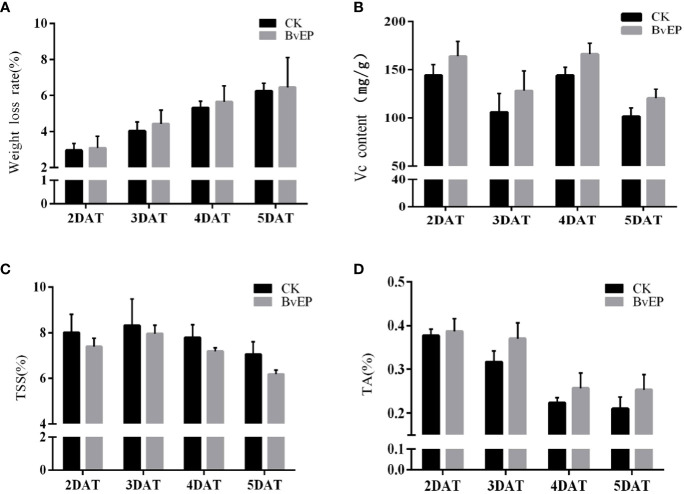
Effect of BvEP on physiological indicators of tomato fruits during storage. **(A)** Weight loss of tomato fruits at 2 DAT, 3 DAT, 4 DAT, and 5 DAT with pK7LIC4.0 and pK7LIC4.0-BvEP; **(B–D)** are separately Vc, TSS, TA content in tomato fruits at 2 DAT, 3 DAT, 4 DAT, and 5 DAT with pK7LIC4.0 and pK7LIC4.0-BvEP.

To verify the effects of BvEP on the quality and physiological indicators of tomato fruits, the postharvest weight loss rate and the content of TSS, TA and Vc were determined. The tomato fruits transformed with the pK7LIC4.0 were used as CK. The results showed that the weight loss, TSS, and Vc contents of tomato fruits 2 DAT, 3 DAT, 4 DAT, and 5 DAT were slightly lower than CK, and TA contents were higher than those of CK, but there was no significant difference (**Figure 7**).

## Discussion

The novel elicitor protein PPM, which can significantly improve tomato resistance to *B. cinerea*, was selected from the *B. velezensis* LJ02 and named BvEP (**Figures 1**, **2**). PPM plays an important role as a key protein in the energy source rescue pathway during nucleoside synthesis in a variety of microorganisms, the pentose portion of nucleosides can be used as carbon source and energy source (Rashid et al., 2004; Tozzi et al., 2006). PPM can participate in the biosynthesis of glucose and RNA, as well as in the pentose phosphate pathway and purine metabolism to obtain carbon sources and energy (Rashid et al., 2004; Tozzi et al., 2006). Carbon source and energy are critical to plant disease resistance (Andolfo and Ercolano, 2015). In addition, sugar signaling can contribute to immune responses against pathogens, interact with hormones such as ABA and SA, and act as initiating molecules to induce PTI and ETI defense pathways in plants (Bolouri Moghaddam and Van den Ende, 2012; Andolfo and Ercolano, 2015). However, there are no reports on the PPM improving of plant disease resistance. In this study, BvEP was overexpressed into tomato leaves by *Agrobacterium* transient transformation, and it was found that BvEP could significantly improve the resistance of tomato leaves to *B. cinerea* (**Figure 2**), indicating that BvEP has a significant effect on the disease resistance of tomato. The purified BvEP was obtained by prokaryotic expression and purification for exploring the reason for disease prevention (**Figures 3A, B**). The result showed that the purified BvEP had no inhibitory effect on the growth of mycelial and spore germination of *B. cinerea in vitro* (**Figures 3C, D**), indicating that BvEP had no direct antibacterial effect on *B. cinerea in vitro*. Therefore, we speculated that BvEP could induce plant systemic resistance to *B. cinerea*. The generation of HR is an important prerequisite for plant to improve systemic resistance to pathogens (Kombrink and Schmelzer, 2001). The purified protein of BvEP was infiltrated in the leaf of *Nicotiana tabacum*, and the normal HR symptom (necrotic spot) was found to form around the infiltrated leaf (**Figure 3E**), indicating that BvEP could induce the plant immune response. However, the signaling pathway involved in BvEP improving plant disease resistance remains unclear.

In order to further verify the biological function and mechanism of BvEP related to disease resistance, BvEP-overexpressing A57 tomato plant was obtained through the genetic transformation of *Agrobacterium*, and BvEP mRNA was stably accumulated (**Figure 4C**). The disease symptom of BvEP-overexpressed transgenic tomato leaves were milder than CK (**Figure 4A**), and the lesion diameter was significantly smaller than CK (**Figure 4B**), indicating that BvEP could significantly improve the resistance of tomato to *B. cinerea*. Plants can recognize the elicitor proteins produced by beneficial microorganisms to induce plant acquired immunity that is controlled by a signaling pathway in PTI, ETI, and SAR (Wang et al., 2011; Ouyang et al., 2015; Naveed et al., 2020). Therefore, the gene expression in PTI, ETI, and SAR in BvEP overexpressing transgenic tomatoes was examined. The expression level of *PTI5* and *WRKY28*, PTI pathway marker genes (Kim et al., 2009; Nguyen et al., 2010), increased significantly (**Figure 4D**). *UDP* and *UDP1* showed high induction only during ETI (Pombo et al., 2014; Chialva et al., 2018), and their expression level increased significantly in transgenic tomato overexpressing BvEP. The SAR is closely related to PTI and ETI (Kombrink and Schmelzer, 2001; Balint-Kurti, 2019; Zhang et al., 2020b). The *PR1a*, SAR pathway marker gene (Wang et al., 2015; Liu et al., 2019) was significantly induced by BvEP (**Figure 4D**). *NPR1*, which is essential for PR gene induction (Wu et al., 2012; Wang et al., 2018), also significantly increased in transgenic tomato overexpressing BvEP (**Figure 4D**). These results demonstrate that the PTI, ETI, and SAR pathways may be involved in the BvEP-induced immune response in tomato. Therefore, it was speculated that BvEP induced the tomato immune signal pathway and improved resistance of tomato to *B. cinerea*. This study provides a new strategy and research direction for tomato disease resistance breeding.

The loss caused by grey mold is one of crucial issues restricting the tomato industry to reduce losses and increase profits (Williamson et al., 2007; Dean et al., 2012; Shao et al., 2021). BvEP was discovered as a novel elicitor protein to improve resistance to *B. cinerea* in tomato. Subsequently, BvEP was transiently transformed and overexpressed in postharvest tomato fruit, and BvEP mRNA was stably accumulated in tomato fruit (**Figure 5C**). BvEP overexpressing tomato fruits exhibited higher resistance to *B. cinerea* compared to CK (**Figures 5A, B**), and the rotting rate and the lesion diameter caused by *B. cinerea* were significantly lower than CK (**Figures 5D, E**), indicating that BvEP had a significant effect in improving the resistance of tomato fruits to *B. cinerea*. The expression of *PTI5*, *WRKY28*, *UDP*, *UDP1*, *PR1a* and *NPR1* genes in postharvest tomato fruits were significantly up-regulated after transient transformation with BvEP (**Figure 6A**), suggesting that BvEP may induce disease resistance signaling pathways such as PTI, ETI and SAR to improve resistance to *B. cinerea* in tomato fruits. In addition, BvEP overexpression could promote ROS accumulation in tomato fruits (**Figure 6B**). ROS not only has a direct antibacterial effect, but also can act as a defense signal molecule to regulate the activation of resistance-related pathways, cause the up-regulation of resistance-related genes and participate in the disease resistance process such as PTI, ETI and SAR (Taheri and Kakooee, 2017; Singh et al., 2021). However, excessive accumulation of ROS can cause damage to plants and make them more susceptible to pathogens (Taheri and Kakooee, 2017). The activation of antioxidant enzyme system, including reductases such as SOD and POD, is a mechanism used by plants to remove excess ROS (Bela et al., 2015). The activities of SOD and POD enzymes were significantly higher in BvEP transiently transformed tomato fruits than CK (**Figures 6C, D**). SOD is the first line of defense against ROS damage, and the increase of POD activity can inhibit the accumulation of reactive oxygen intermediates (ROI) (Ueda et al., 2013; Wagner et al., 2013). In addition, there were no significant differences in weight loss rate and TSS, TA and Vc contents in tomato fruits after transient expression of BvEP compared with CK, indicating that BvEP had little influence on quality related physiological indexes of tomato fruits (**Figure 7**). Improving intrinsic immunity of tomato fruit by exogenous induction is an effective strategy against *B. cinerea* (Zhao et al., 2008; Wang et al., 2011; Zhu and Zhang, 2016). The above results can help to discover new ways of tomato fruit disease resistance and supply a theoretical basis for innovative tomato fruit postharvest green preservation technology.

## Conclusion

In this study, the novel elicitor BvEP was screened to improve the resistance of tomatoes to *B. cinerea*, and further found that BvEP was shown to induce HR response of *Nicotiana tabacum* and could not inhibit *B. cinerea in vitro*. The leaves of transgenic tomatoes overexpressing BvEP can improve the resistance to *B. cinerea* and the expression of PTI, ETI, and SAR pathway marker genes. Moreover, transient overexpression of BvEP could reduce the rotting rate and lesion diameter of tomato fruits caused by *B. cinerea*, increased the expression of resistance genes, ROS content, SOD and POD enzyme activities, while there was no significant effect on the quality related indexes. This study provides a new strategy and research direction for tomato disease resistance breeding.

## Data availability statement

The datasets presented in this study can be found in online repositories. The names of the repository/repositories and accession number(s) can be found in the article/**Supplementary Material**.

## Author contributions

YW, RC and ZL designed the study. ZL, JH, QS and XZ performed the research, analyzed most of the data, and wrote the first draft of the manuscript. YW, RC and ZL contributed to refining the ideas and finalizing this manuscript. YW, RC and ZL wrote the final draft of the manuscript. All authors contributed to the article and approved the submitted version.

## Funding

This research was funded by Tianjin Municipal Education Commission (2021KJ105).

## Acknowledgments

We thank the Scientific Project of Tianjin Municipal Education Commission (2021KJ105) for supporting this work.

## Conflict of interest

The authors declare that the research was conducted in the absence of any commercial or financial relationships that could be construed as a potential conflict of interest.

## Publisher’s note

All claims expressed in this article are solely those of the authors and do not necessarily represent those of their affiliated organizations, or those of the publisher, the editors and the reviewers. Any product that may be evaluated in this article, or claim that may be made by its manufacturer, is not guaranteed or endorsed by the publisher.
