# Supplementary material for: A novel elicitor protein phosphopentomutase from Bacillus velezensis LJ02 enhances tomato resistance to Botrytis cinerea
Source: Front Plant Sci. 2022 Nov 29;13:1064589. doi: 10.3389/fpls.2022.1064589 (PMC9746712; doi:10.3389/fpls.2022.1064589)
Supplement: Supplementary file 1 [file DataSheet_1.pdf]

Supplementary Material

1 Supplementary Figure and Tables

1.1 Supplementary Figure

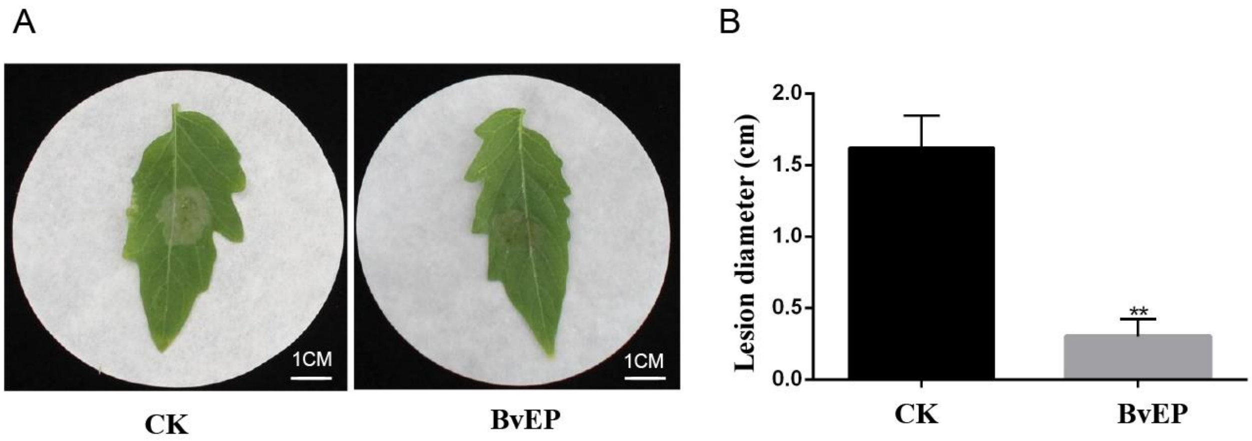

Supplementary Figure 1. Effects of purified BvEP on tomato leaves after inoculation with *B. cinerea*. (A) Phenotype of tomato systemic leaves inoculation at 3 DAI with *B.cinerea*; leaves were treated as follows before inoculation: CK represented Tris buffer infiltration, BvEP represented 50 μg·ml^{-1} purified BvEP infiltration; (B) Lesion diameter of tomato at 3 DAI with *B. cinerea*, asterisks indicate statistically significant differences (** $p < 0.01$).

1.2 Supplementary Tables

Supplementary Table 1: Primers for the identification of BvEP mRNA accumulation in tomato.

Primer ID	Sequence
BvEP-F	CTGTCGGAATCGGTGAAGCG
BvEP -R	GTAGTACGCAAGAGGGTGCTCC

Supplementary Table 2: Specific primers for qRT-PCR of BvEP overexpression in tomato.

Primer ID	Sequence	Reference	Description
Pti5-F	ATTCGCGATTCGGCTAGACATGGT	Liu et al., 2012	Detection of tomato fruits and leaves
Pti5-R	AGTAGTGCCTTAGCACCTCGCATT	Liu et al., 2012	
WRKY28-F	ACAGATGCAGCTACCTCATCCTCA	Liu et al., 2012	
WRKY28-R	GTGCTCAAAGCCTCATGGTTCTTG	Liu et al., 2012	
PR1a-F	GGCAGGAACACCAAAGAAACCA	Zhang et al., 2020	
PR1a-R	TGGCCTCTGGTCAGGTTTAAAG	Zhang et al., 2020	
NPR1-F	GACTTCTTCGCTGATGCTAAGC	Sun et al., 2013	
NPR1-R	GACCACGGCATCAAAACTCACC	Sun et al., 2013	
Actin-F	CCAGGTATTGCTGATAGAATGAG	Zhang et al., 2020	
Actin-R	GAGCCTCCAATCCAGACAC	Zhang et al., 2020	
UDP-F	CCTGGATTGTTCGACAAGAT	Pombo et al., 2014	Detection of tomato fruits
UDP-R	CTCCTCCGCTTTCTTCATTT	Pombo et al., 2014	
UDP1-F	TTGGACAGATCAAGGGACTAATG	Pombo et al., 2014	
UDP1-R	CACTCTCAACCACACCATCTT	Pombo et al., 2014	
UDP-F	CAAAGCTGAAAGAGGGAACG		Detection of tomato leaves
UDP-R	TAACCCAAGCCCTAGCTCAAC		
UDP1-F	TTTGAAAGGGTCCCAAATCC		
UDP1-R	TGCAGAGGGGTGTCAATTTC		
